# A Case of Necrotizing Lymphadenitis From Streptococcus pyogenes

**DOI:** 10.7759/cureus.84673

**Published:** 2025-05-23

**Authors:** Brittani P Kongala, David J Gorelov, Anwar A Khan, Tetyana Shvets, Ashley Falk

**Affiliations:** 1 Medicine, Florida State University College of Medicine, Tallahassee, USA; 2 Family Medicine, Florida State University College of Medicine, Winter Haven, USA

**Keywords:** bacterial lymphadenitis, cervical lymphadenitis, cervical lymph node, group a streptococcus pyogenes, kikuchi-fujimoto disease, necrotizing lymphadenitis

## Abstract

Necrotizing lymphadenitis (NL) is a rare occurrence of necrotic, nongranulomatous lymphadenopathy. While idiopathic, autoimmune, and viral etiologies are commonly implicated in NL, bacterial etiologies, particularly from *Streptococcus pyogenes* (*S. pyogenes*), are exceedingly rare. We present a case of a 25-year-old female who presented with a one-week history of right-sided neck pain, fever, and sepsis. Examination and lab tests revealed an enlarged cervical lymph node measuring 2.4 cm and a positive *Streptococcus* A test. Four days later, the lymph node increased in size to 3.6 cm despite antibiotic management. Due to concerns of abscess formation and compression of the internal jugular vein, the patient underwent ultrasound-guided lymph node aspiration and biopsy, which revealed purulent fluid and necrosis. In our literature search, only one other case of NL secondary to *S. pyogenes* has been described, thus highlighting the clinical significance of this presentation. This case emphasizes the complexity of addressing and treating NL and underscores the need to consider bacterial etiologies in the diagnosis.

## Introduction

Necrotizing lymphadenitis (NL) is a broad term for a group of diseases characterized by necrosis and nongranulomatous inflammation of the lymph node [1,2]. It was originally reported in Japan in the early 1970s and was thought to be a reactive hyperplasia of the lymph node, indicating the lymphadenopathy was in response to an antigen and not of neoplastic origin [2-4]. The condition most commonly affects females and presents with an acute onset of unilateral cervical lymphadenopathy, fever, and odynophagia [1,5,6]. Histopathology of affected lymph nodes reveals large areas of necrosis in the paracortical areas with increased numbers of immunoblasts and macrophages [4].

NL is clinically significant because several diseases, ranging from self-limited to neoplastic, can lead to its development. The most common etiology is a self-limited condition called Kikuchi-Fujimoto disease, also known as histiocytic NL (HNL) [2,7,8]. HNL is idiopathic and characterized on histology by a prominent histiocyte infiltrate accompanied by cervical lymphadenitis and fever [7,8]. It is usually seen in young adult females of Asian descent [2,7,8]. Other etiologies of NL include granulomatous inflammation, metastatic disease, and autoimmune etiologies, such as systemic lupus erythematosus (SLE) [3,4,7]. Infectious causes are less common but are usually due to Epstein-Barr virus (EBV), rubella, paramyxovirus, parainfluenza, *Yersinia enterocolitica*, or *Mycobacterium tuberculosis* [5]. Parasitic etiologies such as *Leishmania major* have also been reported [9,10].

However, bacterial etiologies of NL are rare and usually arise from a preceding cervical lymphadenitis from *Staphylococcus aureus* (*S. aureus*) [1,4]. *Streptococcus pyogenes* (*S. pyogenes*) is an exceptionally rare causative agent of NL, with few documented cases, and is the primary etiology discussed in this clinical report [1]. In our PubMed search from 1970 to present, using the search terms “necrotizing lymphadenitis” or “lymphadenitis” and “streptococcus” or “group A streptococcus”, only one other case of NL secondary to *S. pyogenes* has been reported [1].

## Case presentation

**Figure 1:**
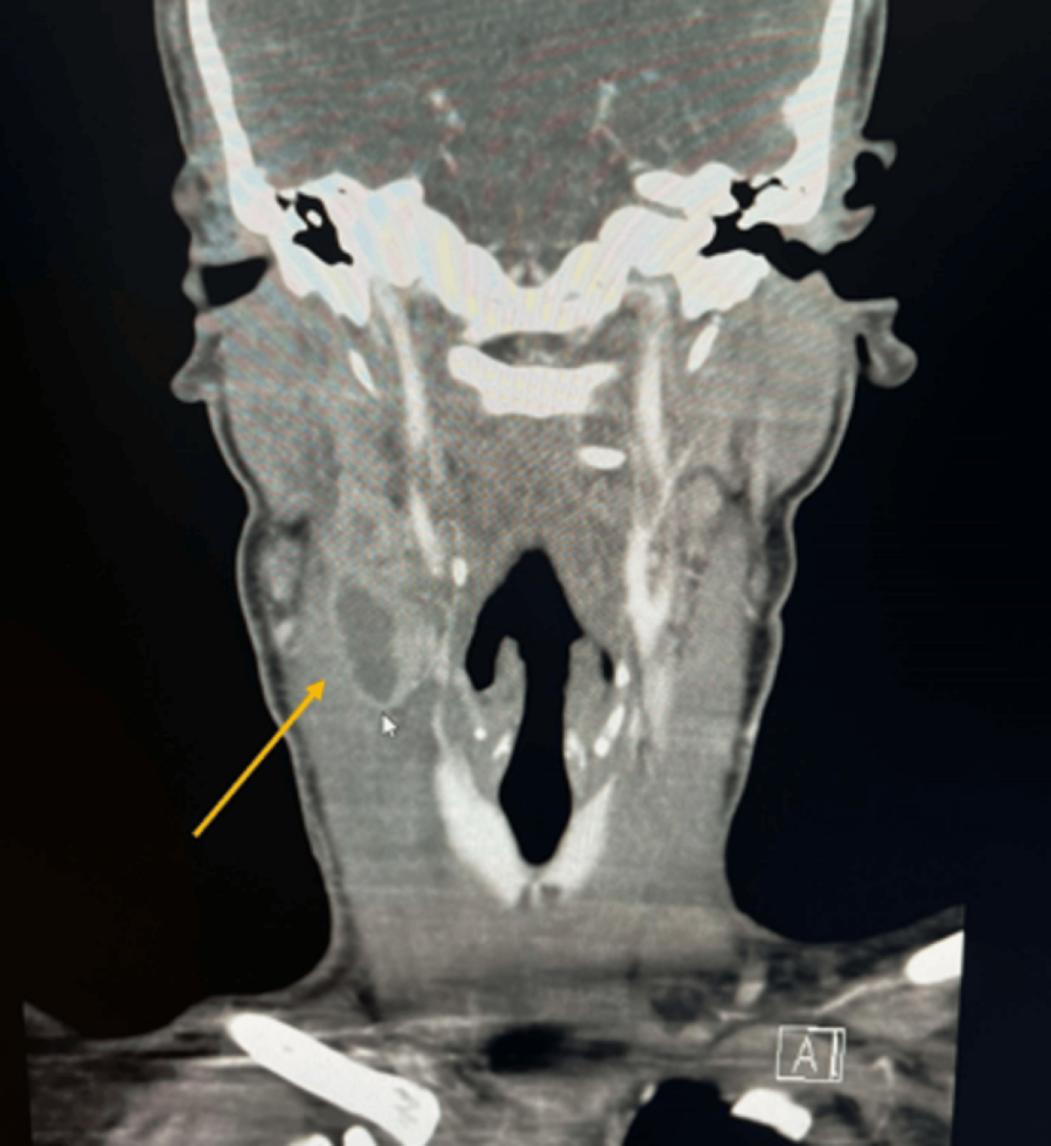
Initial CT on admission showing a necrotic mass measuring 2.4 x 1.7 cm in the right level 2 cervical chain with adjacent sternocleidomastoid myositis and mass effect on the internal jugular vein.

A 25-year-old female with no significant past medical history presented to the emergency department (ED) with a one-week history of right-sided neck pain that worsened with movement, fever, body aches, dysphagia, nausea, and diarrhea. She denied any previous trauma. Her vital signs were as follows: blood pressure of 112/77 mmHg, heart rate of 126, respiratory rate of 18, and temperature of 100.8°F. Physical examination revealed an enlarged and tender right cervical lymph node. Laboratory results indicated an elevated WBC count of 18,000 cells/μL (normal range is 4,000-11,000 cells/μL) and an elevated CRP of 11.28 mg/L (normal is less than 3 mg/L). All other lab values were within reference range. Thyroid, liver, and kidney function tests were within normal ranges. The patient tested positive for *S. pyogenes* via a rapid Strep test and tested negative for influenza, COVID-19, respiratory syncytial virus, EBV, HIV, and hepatitis C. CT imaging revealed a 2.4 x 1.7 cm enlarged lymph node with necrotic changes in the right cervical region with adjacent sternocleidomastoid (SCM) myositis and mass effect on the internal jugular vein (IJV). These findings raised concerns for the potential development of Lemierre’s syndrome, a condition of septic thrombophlebitis in the IJV. The CT image is shown in Figure 1.

**Figure 2:**
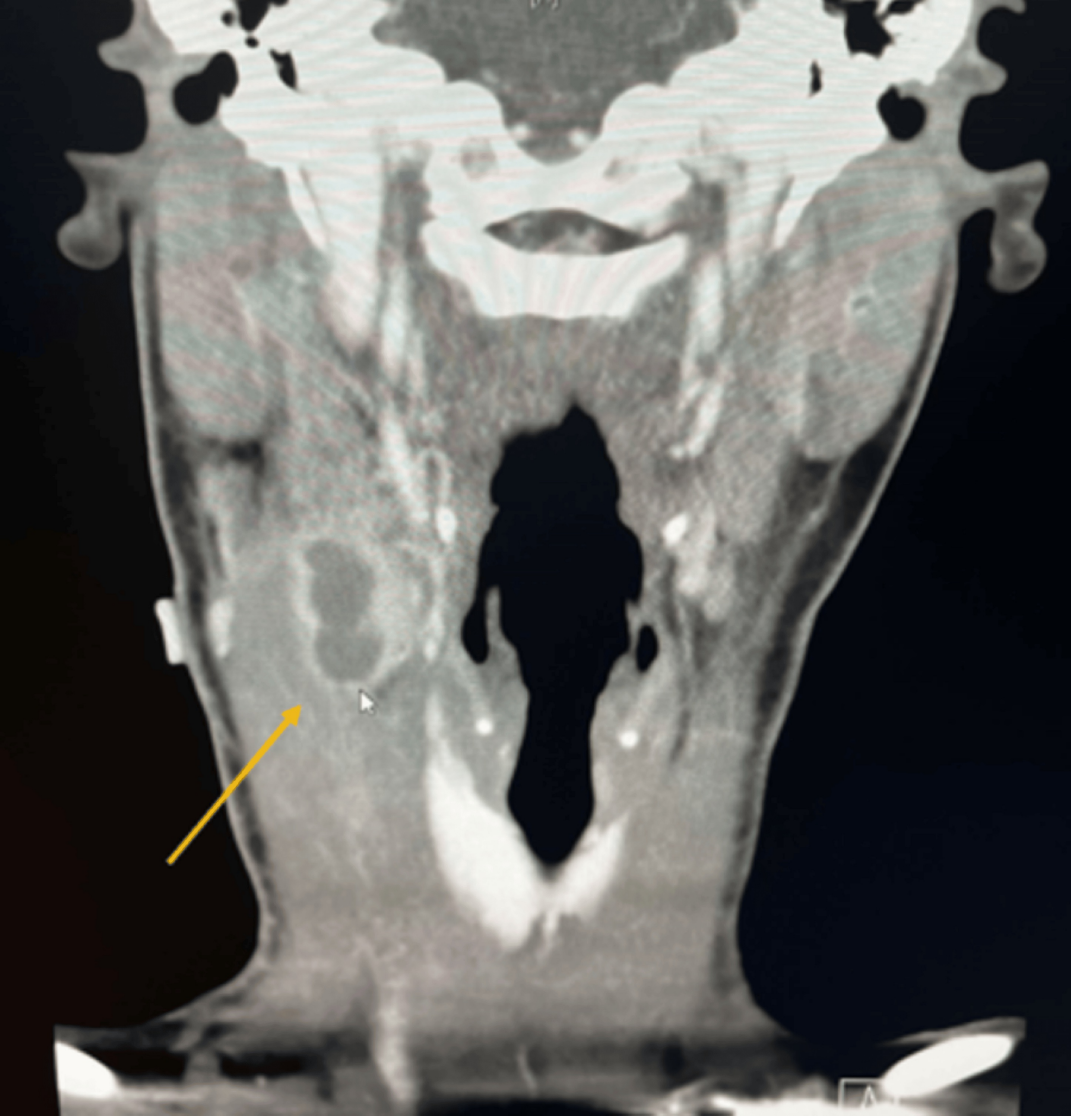
Follow-up CT imaging on day four showing a necrotic mass measuring 3.6 x 1.7 cm in the right cervical chain with adjacent sternocleidomastoid myositis and mass effect on the internal jugular vein.

On presentation to the ED, medical management was initiated with IV clindamycin and dexamethasone. However, by day two of admission, the patient did not show clinical improvement, so she was switched to IV ampicillin/sulbactam with discontinuation of dexamethasone. The patient was closely monitored for possible complications. Despite antibiotic treatment, follow-up CT imaging on day four of admission revealed an increase in lymph node size to 3.6 x 1.7 cm. Figure 2 shows the CT imaging from day four. Continued inflammation of the SCM and compression of the IJV were noted.

Despite medical management, there was a concern for abscess formation as evidenced by the continued enlargement of the lymph node. The patient was also still febrile with temperatures ranging from 101.1°F to 102.8°F. This prompted the use of ultrasound-guided lymph node aspiration, which yielded 6 mL of necrotic, purulent fluid. Subsequent culture of the fluid grew *S. pyogenes*, confirming the diagnosis of NL from an infectious etiology. Post aspiration, the patient reported an improvement in her pain and dysphagia, but continued to have mild tenderness around the affected lymph node.

**Figure 3:**
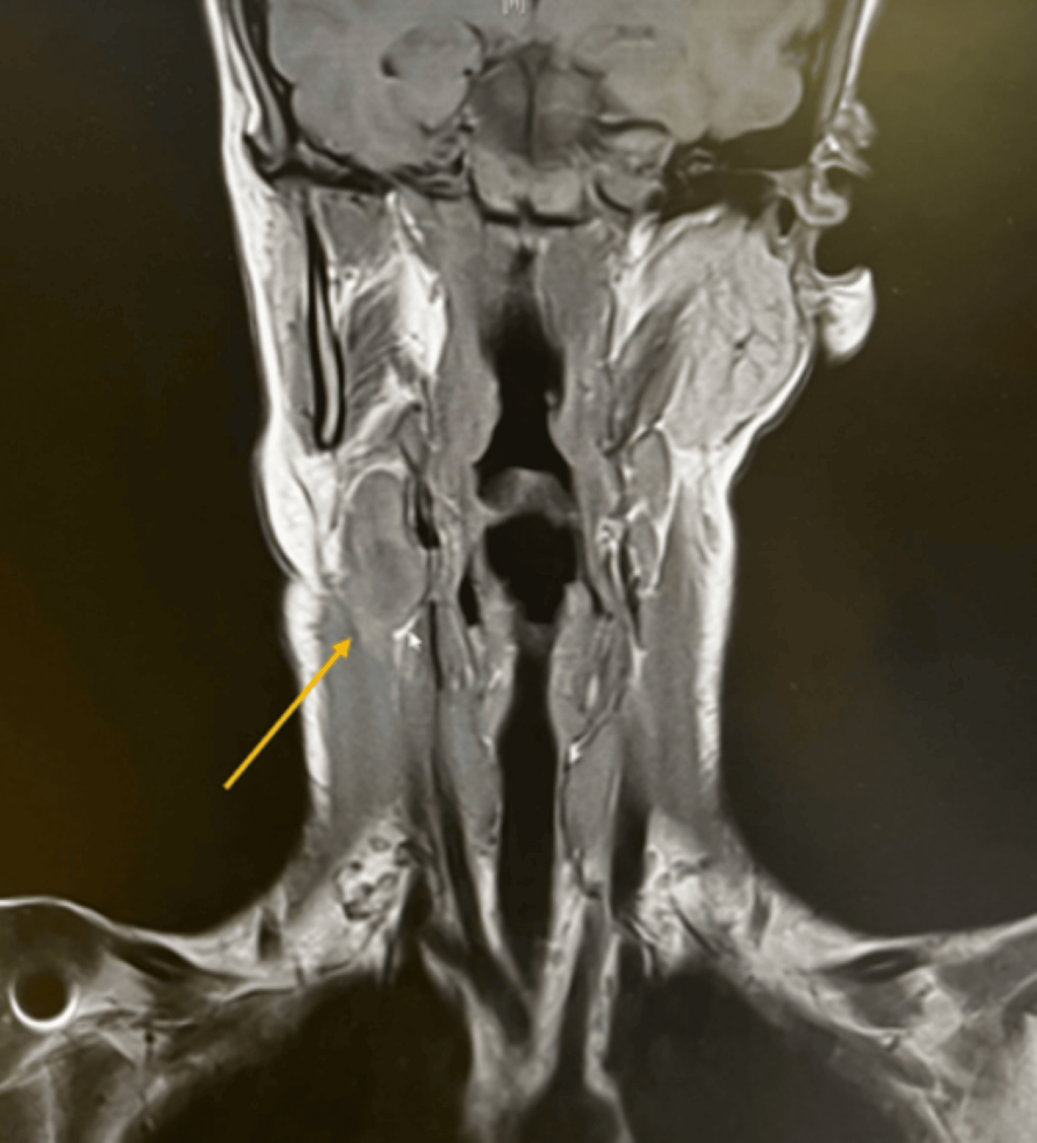
Follow-up MRI one day after ultrasound-guided drainage showing no recollection of fluid.

Treatment with IV ampicillin/sulbactam was continued. In this case, antibiotic susceptibility testing was not performed on the aspirated fluid. As penicillin is the antibiotic of choice for the treatment of infections caused by *S. pyogenes*, the IV ampicillin/sulbactam was continued. A follow-up MRI the day after drainage showed no fluid recollection in the lymph node. Figure 3 shows the MRI post drainage. An MRI was preferred in this instance over CT imaging because of its enhanced tissue visibility. There was great concern in this case regarding the compression of the IJV. MRI was used due to its superior soft tissue contrast, precise anatomical detailing, and enhanced ability to differentiate soft tissue structures for accurate evaluation.

Because the patient was stable and the lymph node had not re-enlarged post drainage, she was discharged with 10 days of oral ampicillin/sulbactam and asked to follow up with an outpatient ENT provider. Four weeks later, at a follow-up appointment, the patient was continuing to improve. She reported a continued decrease in the size of the affected lymph node since her discharge. There was no recurrence of lymph node enlargement, and her pain and dysphagia had resolved.

## Discussion

While previously reported predominantly in Asia, NL is increasingly recognized worldwide, making it a clinically relevant condition for practitioners globally [1,3,11]. Due to its low prevalence, it is often misdiagnosed as tuberculosis or lymphoma, both of which can present with NL [1,3,7]. As such, tuberculosis and lymphoma remain important differential diagnoses for NL and must be excluded using histopathology [2,7]. Another differential for NL is SLE. Most cases of NL occur in young women and often correlate with chronic inflammatory conditions like SLE [1-3,6]. However, in the present case, autoimmunity was excluded based on clinical signs of infection, leukocytosis, and abscess formation within the lymph node. The patient also failed to show any clinical improvement with the initiation of dexamethasone. The patient’s positive rapid streptococcal antigen test, along with the isolation of *S. pyogenes *from aspirated fluid, confirmed a bacterial etiology rather than a reactive lymphadenopathy. Viral etiologies were also excluded based on the negative EBV, HIV, and hepatitis C testing. HNL is another possible differential diagnosis for NL. However, in this case, the culture of *S. pyogenes* is consistent with a bacterial infection rather than an idiopathic progression.

The diagnosis of NL is primarily based on the clinical presentation and the histopathological findings from lymph node biopsy, which reveals enlarged cervical lymph nodes with large areas of non-granulomatous necrosis [2,3,12]. The normal lymph node structure is replaced by an irregular shape with sheet-like foci of coagulation in the paracortical regions [3,13]. The necrotic foci consist of disintegrating cellular debris with necrotic vascular remnants and hemorrhage [3]. Apoptotic bodies are found surrounding the area of necrosis [3]. In addition, monocytes and immunoblasts can be visualized infiltrating the tissue [1,3]. In cases of NL arising from bacterial infection, neutrophils can also be seen infiltrating the affected lymph node [14]. This is a key differentiating factor from HNL, which presents with a histiocyte infiltrate and notable lack of neutrophils.

CT imaging can also be used to confirm the diagnosis of NL [13]. The affected lymph nodes will display homogenous contrast enhancement or hypoattenuation of necrotic areas with surrounding cortical enhancement, as was seen in this case [13]. Fine-needle aspiration (FNA) may be pursued if an infectious etiology is suspected. The most common bacterial causes of NL are *S. aureus* and *Mycobacterium tuberculosis* [3,5,11]. However, reports of NL secondary to *S. pyogenes* are exceedingly rare, with only one other case discovered in our literature search [1]. Therefore, our case is clinically significant as it highlights an exceptionally rare etiology of NL in an immunocompetent individual.

While *S. pyogenes* is a recognized cause of cervical lymphadenitis, progression to NL is an uncommon clinical outcome and may be underreported in the literature [4]. Cervical lymphadenitis is an inflammation and infection of the cervical lymph nodes that is usually caused by acute viral or bacterial etiologies [15]. It is characterized by unilateral or bilateral enlargement of the cervical lymph nodes and is most commonly seen in children. *S. pyogenes* and *S. aureus* are the most frequent etiologies [1,4]. NL is a rare complication of bacterial lymphadenitis [1]. While the precise pathophysiology of NL secondary to bacterial infection, particularly from *S. pyogenes*, has historically been obscure, recent research suggests a potential mechanism [16]. It is likely that *S. pyogenes* directly invades cervical lymph nodes via hematogenous or lymphatic spread, resulting in suppuration and necrosis [1,4,16]. It is thought that *S. pyogenes* can utilize afferent lymphatics to invade local draining lymph nodes from primary infection sites, similar to the mechanism of neoplastic metastasis [16]. These invasive strains of *S. pyogenes* are believed to possess genetic mutations that enhance the bacteria’s virulence factors and enable the proliferation of the bacteria within the lymph node, contributing to the severe inflammatory and destructive process observed in NL [16].

In our case, the patient also developed unique complications associated with this infectious etiology of NL, which have not been documented. Specifically, the patient developed SCM myositis and mass effect on the IJV, demonstrating how locally advanced NL can lead to potentially severe complications if not managed promptly. Due to the proximity of cervical lymph nodes to vital anatomical structures, complications from NL can include dysphagia, airway compromise due to mass effect, bacteremia, septic thrombophlebitis, necrotizing fasciitis, and mediastinitis, all of which may necessitate intensive interventions, such as surgical debridement [10].

Given its rarity, particularly in the United States, the diagnosis and management of bacterial NL present a challenge [1]. Clinicians should maintain a high index of suspicion for NL in patients, especially young females, who present with upper respiratory symptoms, cervical lymphadenopathy, and a neck mass. Early recognition, as demonstrated in this case, is crucial for timely diagnosis and appropriate management. However, despite early recognition and the initiation of appropriate antibiotic therapy with IV ampicillin/sulbactam, in this case, the patient’s lymphadenopathy remained refractory to treatment. This emphasizes the importance of serial imaging to monitor disease progression. Ultimately, ultrasound-guided FNA, which was both diagnostic and therapeutic, was required in combination with continued IV ampicillin/sulbactam to resolve the patient’s NL. FNA is a reliable, safe procedure to isolate the causative organism and determine antibiotic therapy in cases of bacterial infection [4]. This is consistent with the existing literature, which suggests that aspiration or surgical drainage is necessary in cases of enlarging or refractory lymphadenopathy after initial medical management proves insufficient [4,17].

## Conclusions

NL is a rare but severe form of lymphadenopathy that is usually preceded by cervical lymphadenitis when it is of bacterial etiology. While viral and mycobacterial etiologies are more commonly considered, bacterial pathogens, especially *S. pyogenes*, should remain on the differential, particularly when suppuration is evident. This case highlights the critical importance of the early recognition of NL and close monitoring with serial imaging. While selecting the appropriate antimicrobial therapy is critical, drainage should be considered for refractory lymphadenopathy to ensure optimal patient outcomes.
